# *Trichinella spiralis* co-infection exacerbates *Plasmodium berghei* malaria-induced hepatopathy

**DOI:** 10.1186/s13071-020-04309-6

**Published:** 2020-09-03

**Authors:** Xu Mei, Zhanhong Ye, Yuqing Chang, Shiguang Huang, Jianping Song, Fangli Lu

**Affiliations:** 1 Artemisinin Research Center and Institute of Science and Technology, Guangzhou University of Chinese Medicine, Guangzhou, China; 2 Department of Parasitology, Zhongshan School of Medicine; Key Laboratory of Tropical Disease Control of Ministry of Education, Sun Yat-sen University, Guangzhou, China; 3 School of Stomatology, Jinan University, Guangzhou, China

**Keywords:** Mice, *P. berghei*, *T. spiralis*, Co-infection, Liver pathology, Galectins, Macrophages, Neutrophils, Eosinophils

## Abstract

**Background:**

Although *Plasmodium* parasites and intestinal helminths share common endemic areas, the mechanisms of these co-infections on the host immune response remain not fully understood. Liver involvement in severe *Plasmodium falciparum* infections is a significant cause of morbidity and mortality. However, the effect of pre-existing *Trichinella spiralis* infection on the immune response and liver immune-pathogenesis in *P. berghei* ANKA (*Pb*ANKA)-infected mice needs to be elucidated.

**Methods:**

Outbred Kunming mice were infected with *T. spiralis* and 9 days later were challenged with *P. berghei* ANKA (*Pb*ANKA), and the investigation occurred at 13 days after co-infection.

**Results:**

Compared with *Pb*ANKA-mono-infected mice, *T. spiralis* + *Pb*ANKA-co-infected mice had similar survival rate but lower *Pb*ANKA parasitaemia; however, there were more severe hepatosplenomegaly, increased liver and spleen indexes, and increased liver pathology observed by hematoxylin and eosin staining; higher expression levels of galectin (Gal)-1, Gal-3, CD68^+^ macrophages, and elastase-positive neutrophils measured by immunohistochemical staining; upregulated mRNA expression levels of Gal-1, Gal-3, cytokines (interferon-gamma (IFNγ) and interleukin (IL)-6), and M1 macrophage polarization marker (inducible nitric oxide synthase (iNOS)) in the liver, and increased expression levels of Gal-1, IFNγ, IL-6, eosinophil cationic protein, eosinophil protein X, and M1 (IL-1β and iNOS) and M2 (Ym1) macrophage polarization markers in the spleen of co-infected mice detected by using quantitative real-time reverse transcription polymerase chain reaction (qRT-PCR). *In vitro* study showed that compared with *Pb*ANKA-mono-infected mice, there were significantly increased expression levels of Gal-1, Gal-3, IL-6, IL-1β, and iNOS in the peritoneal macrophage isolated from co-infected mice detected by using qRT-PCR. Correlation analysis revealed significant positive correlations between Gal-3 and IL-1β in the peritoneal macrophages isolated from *Pb*ANKA-mono-infected mice, between Gal-3 and IFNγ in the spleen of co-infected mice, and between Gal-1 and Ym1 in the peritoneal macrophages isolated from co-infected mice.

**Conclusions:**

Our data indicate that pre-existing infection of *T. spiralis* may suppress *P. berghei* parasitaemia and aggravate malaria-induced liver pathology through stimulating Gal-1 and Gal-3 expression, activating macrophages, neutrophils, and eosinophils, and promoting mediator release and cytokine production.
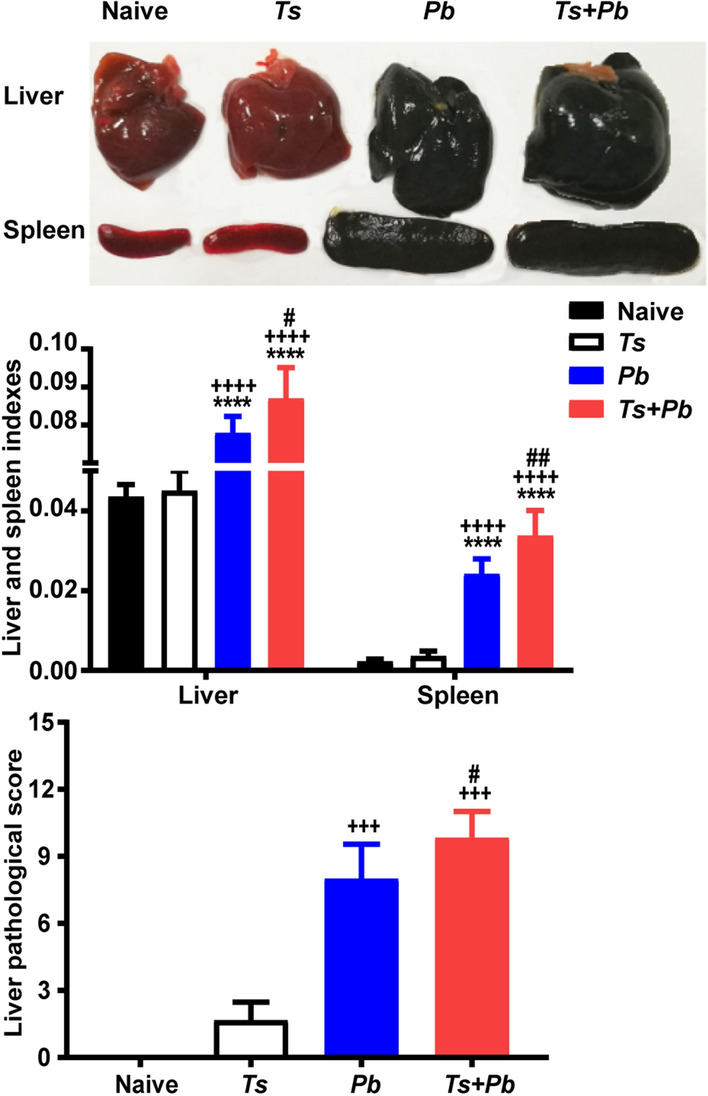

## Background

The manifestations of severe malaria often present clinically as cerebral malaria, pulmonary edema, acute kidney injury, hypoglycaemia, lactic acidosis, anemia, and liver involvement [1]. Helminths and malaria are among the most prevalent infectious diseases in the world [2]. Malaria remains a serious public health issue in sub-Saharan region, while soil-transmitted helminths, such as *Ascaris lumbricoides*, *Trichuris trichiura*, and the hookworm species, infect more than a billion people worldwide [3], which have wide geographical overlap with malaria prevalent areas [4]. However, how concurrent infections of parasites affect the epidemiology and pathogenesis of each other remains controversial. It has been reported that the incidence rate of malaria attacks was significantly higher among *Schistosoma mansoni*-infected patients [5]. Mixed *Plasmodium falciparum* and *P. vivax* infections were found to be more frequent in *A. lumbricoides*-infected patients in Thailand, while infection with *A. lumbricoides* was associated with a dose-dependent effect on protection from cerebral malaria and acute renal failure [6]. Furthermore, a controlled randomized trial of anti-helminthic treatment showed a negative interaction between *A. lumbricoides* and *P. falciparum* malaria parasite density in Malagasy population (over 5 years-old), yielded to a higher malaria transmission [7]. However, in southern Ethiopia, *T. trichiura* infection was associated with increased malaria prevalence, while an increased worm burden of helminths as expressed by egg intensity was associated with increased malaria parasitaemia [4]. In addition, intensity of hookworm infection was associated with increased *P. falciparum* or *P. vivax* parasitemia [8]. In Uganda, *P. falciparum* and *Taenia* spp. co-infections constitute an important risk to anemia and malaria in the co-infected children [9]. It has been shown that heavy *A. lumbricoides* and *T. trichiura* infections were associated with high *P. falciparum* parasitaemia in a peri-urban community in Kwara State, Nigeria [10].

*Trichinella spiralis* can infect humans and more than 150 types of other animals *via* eating raw or undercooked pork, causing trichinellosis, a globalized zoonotic parasitic disease [11]. Cases of trichinellosis have been reported worldwide except for Antarctica [12, 13]. *Trichinella spiralis* infection in rodents has been used extensively to study the impact of inflammation on the small intestine [14]. *Trichinella spiralis* infection can induce an increase of small intestine mucosal immune cells, and the intestinal mucosal immune system of infected mice was induced toward mixed Th1/Th2 phenotypes with the predominance of Th2 response at the early stage of infection [15]. Arrival of newborn larvae in muscle is coincident with an intestinal Th2 immune response that expels adult worms and induces prominent blood and tissue eosinophilia [16].

So far, the impact of helminths on liver immunopathology induced by malaria is not well-understood. Pre-*T. spiralis*-infected animal model has been used to study Th2 immune response on malaria by some researchers [17, 18]. Therefore, in the present study we established a mouse model co-infected with the rodent blood-stage malaria parasite *P. berghei* of strain ANKA (*Pb*ANKA) and a pre-existing nematode *T. spiralis* to assess the influence of concurrent helminth parasite infection on the development of malarial hepatopathy. We found that co-infection with *T. spiralis* increased the immune response and exacerbated *Pb*ANKA malaria-induced liver immunopathology by altering a number of key immune factors.

## Methods

### Mice, parasites, and infections

Female, 6–8 weeks-old Kunming mice were purchased from the animal facility at Sun Yat-sen University in Guangzhou, China. *Trichinella spiralis* (pig strain) was maintained in our laboratory *via* serial passage in mice, and the larvae were recovered from muscles of mice at 60–90 days post-infection (pi). Standard procedures were used for isolation, collection, and inoculum [19]. *Pb*ANKA parasites were maintained at liquid nitrogen as a cryo-frozen stock of parasitized red blood cells (pRBCs) in our laboratory. The parasites were prepared through 3 cycles of passage of the pRBCs in mice. The experimental mice were each infected with 1 × 10^6^ pRBCs by intraperitoneal (ip) injection. A total of 70 mice were used in this experiment. Mice were randomized into 4 groups; except for the uninfected control group consisting of 10 mice, the other 3 groups consisted of 20 mice per group. *Trichinella spiralis* infection was performed 9 days prior to *Pb*ANKA infection: (i) uninfected control group; (ii) *T. spiralis-*infected group: mice were mono-infected with 20 *T. spiralis* larvae by oral gavage on day 0 that was assumed as the day of *T. spiralis* inoculation; (iii) *Pb*ANKA-infected group: mice were mono-infected with 1 × 10^6^ *Pb*ANKA in 0.1 ml of PBS administered by ip injection; and (iv) co-infected group: mice were infected with 20 *T. spiralis* and 9 days later challenged with 1 × 10^6^ *Pb*ANKA. Mortality of each mouse was monitored daily, body weight was monitored from day 4 pi, and parasitemia was monitored daily by Giemsa-stained thin blood smears of tail blood. Erythrocyte counts were performed with a hematocytometer, and more than 1000 RBCs were counted under 1000× oil-immersion light microscopy to determine the percentage of pRBCs [20].

### Measurement of serum enzyme activities

Mice were euthanized in a chamber by CO_2_ asphyxiation on day 22 after *T. spiralis* infection and/or on day 13 after *Pb*ANKA infection. The blood samples of mice were collected and centrifuged at 800×*g* for 10 min. After centrifugation, the supernatant was collected and stored at −20 °C until further use. Serum levels of aspartic aminotransferase (AST) and alanine aminotransferase (ALT) were measured by different assay kits (ALTL/ASTL; Roche, Mannheim, Germany) to determine the liver damage using an automatic biochemical analyzer (Roche-Hitachi cobas 8000 c702 Chemistry Autoanalyzer; Roche Diagnostics, Tokyo, Japan) according to the manufacturer’s instructions.

### Determination of the liver and spleen indexes

On day 22 after *T. spiralis* infection and/or on day 13 after *Pb*ANKA infection, the body weight of each mouse was measured before sacrificed, and the livers and spleens of mice were excised and weighed. Liver index = liver weight (g)/body weight (g) × 100%, and spleen index = spleen weight (g)/body weight (g) × 100%.

### Histopathological analyses

Mice were euthanized by CO_2_ asphyxiation on day 22 after *T. spiralis* infection and/or on day 13 after *Pb*ANKA infection, and livers and spleens were harvested and immediately fixed in 10% buffered natural formaldehyde (Guangzhou Chemical Reagent Factory, Guangzhou, China) for over 48 h. Four-micrometer-thick serial tissue sections of the organs from each mouse were stained with hematoxylin and eosin (H&E) (Sigma-Aldrich, St. Louis, MO, USA) and imaged under light microscopy. To evaluate the hepatic histological alteration, a semi-quantitative scoring system was used. The liver lesions were scored for 4 parameters as previously described, including (i) architecture loss, (ii) pRBCs in microvessels, (iii) pigment deposition, and (iv) portal tract inflammation [21, 22]. The liver damage score ranged from 0 to 3 (0, normal; 1, mild; 2, intermediate; and 3, most serious). Score 0 meant no histopathological change and score 12 referred to the most severe histopathological change. Scoring of each tissue sample represented the mean score of at least five separate microscopic fields at a magnification of 400×.

### Immunohistochemistry

After the liver and spleen tissue sections (4 µm) were deparaffinized and rehydrated in distilled water, heat-induced antigen retrieval was performed in citrate buffer in an 800 W microwave oven for 30 min. Sections were treated with 3% hydrogen peroxide in methanol for 10 min at room temperature to inactivate endogenous peroxidase, and then incubated in 10% bovine serum albumin in phosphate buffered solution (PBS, pH 7.4) for 60 min at room temperature to block non-specific binding. After washing with PBS, sections were incubated with the primary antibodies including CD68 (1:200, Bsm-33056m; Beijing Biosynthesis Biotechnology Co., Ltd., Beijing, China), neutrophil elastase (1:100, PB1114; Wuhan Boster Biological Engineering Co., Ltd., Wuhan, China), galectin (Gal)-1 (1:250, A00470; Wuhan Boster Biological Engineering Co., Ltd.), or Gal-3 (1:200, Bs-0721r; Beijing Biosynthesis Biotechnology Co., Ltd.) at 37 °C for 1 h. Negative controls were performed without a primary antibody. Then, slides were incubated with secondary antibody (goat anti-mouse/rabbit IgG, PV-9001 kit; Beijing Zhongshan Golden Bridge Biotechnology Co., Ltd., Beijing, China). Antigens were visualized using a DAB kit (ZLI-9017; Beijing Zhongshan Golden Bridge Biotechnology Co., Ltd.). Four non-repeating immunohistochemical images of each sample were visualized and acquired using Olympus BX63 microscope image system (Olympus, Tokyo, Japan) under high power field (magnification of 400×). The immunohistochemical signals (positive areas) were quantified using images captured with a digital camera system and analyzed by using Image-Pro Plus software (Image Z1 software, version 6.0; Media Cybernetics, Silver Spring, MD, USA).

### Isolation of murine peritoneal macrophages

Following *T. spiralis* infection on day 22 pi and/or *Pb*ANKA infection on day 13 pi, naive mice, *T. spiralis-*infected mice, *Pb*ANKA-infected mice, and *T. spiralis* + *Pb*ANKA-co-infected mice were ip injected with 2 ml of 3% thioglycollate broth (Sigma-Aldrich) once daily for 3 days. Animals were sacrificed and their peritoneal macrophages were harvested by peritoneal wash with 2 ml PBS containing 10% fetal calf serum (FBS), which was repeated 3 times. Peritoneal lavage fluid was centrifuged at 800×*g* at 4 °C for 5 min, and the pelleted peritoneal macrophages were re-suspended in RPMI 1640 culture medium containing 10% FBS and seeded at 5 × 10^5^ cells/well in 12-well plates (Corning, NY, USA) at 37 °C in a 5% CO_2_ atmosphere. After 2 h, the non-adherent cells were removed, and adherent cells were washed and collected. Samples were stored at −80 °C until subjected to further mRNA analysis.

### Determination of mRNA expression by using quantitative real-time reverse transcription-polymerase chain reaction (qRT-PCR)

Total RNA was extracted from about 100 mg of liver and spleen tissues and peritoneal macrophages isolated from each mouse using an RNA extraction kit (TaKaRa, Shiga, Japan) as per the manufacturer’s protocol. RNA amount was determined by measuring the ratio of absorbance at 260 and 280 nm using a NanoDrop ND-1000 spectrophotometer (NanoDrop Technologies, Wilmington, USA). First-strand cDNA was constructed from 1.0 µg of total RNA with oligo(dT) as primers using a PrimeScript 1st Strand cDNA Synthesis Kit (TaKaRa). To determine tissue mRNA levels of Gal-1, Gal-3, cytokines (IFNγ, IL-1β, IL-6, iNOS, and Ym1), C-C motif chemokine ligand 11 (CCL11), CCL24, eosinophil cationic protein (ECP), and eosinophil protein X (EPX), qRT-PCR measurements were performed using SYBR Green QPCR Master Mix (TaKaRa). Primers are listed in Table 1. Briefly, a total of 10 µl reaction mixture contained 5.0 µl of SYBR® Premix Ex TaqTM (2×), 0.5 µl of each primer (10 pM), 3.0 µl of dH_2_O, and 1.0 µl of cDNA (0.2 µg/µl). Amplification was pre-denaturized for 30 s at 95 °C, followed by 43 cycles of 5 s at 95 °C and 20 s at 60 °C with a LightCycler® 480 instrument (Roche Diagnostics). The results are expressed as fold change compared with uninfected controls.

**Table 1:** Primer sequences of genes used for quantitative real-time reverse transcription-polymerase chain reaction assays

Gene	Forward primer (5′-3′)	Reverse primer (5′-3′)	GenBank ID
GAPDH	CAACTTTGGCATTGTGGAAGG	ACACATTGGGGGTAGGAACAC	NC_000072.6
Gal-1	GCCTACACTTCAATCCTCGCT	GTTCCCGGTGTTCGGTTCC	NM_008495
Gal-3	GGAGAGGGAATGATGTTGCCT	TCCTGCTTCGTGTTACACACA	NM_001145953
CCL11	GAATCACCAACAACAGATGCAC	ATCCTGGACCCACTTCTTCTT	NC_000077.6
CCL24	ATTCTGTGACCATCCCCTCAT	TGTATGTGCCTCTGAACCCAC	NM_019577
ECP	ATCCAAGTGGCTTGTGCAGTGAC	TAAGGTGTTCTCCTCCGACTGGTG	NC_000080.6
EPX	CTCACCCAACACGCTGAAG	TTTTCTGTGTGTGATTGTAGGCA	NM_007946
IFNγ	ATGAACGCTACACACTGCATC	CCATCCTTTTGCCAGTTCCTC	NM_008337
IL-1β	GCAACTGTTCCTGAACTCAACT	ATCTTTTGGGGTCCGTCAACT	NM-008361
IL-6	CCGGAGAGGAGACTTCACAG	CATTTCCACGATTTCCCAGA	NC_000071.6
iNOS	GTTCTCAGCCCAACAATACAAGA	GTGGACGGGTCGATGTCAC	NM_010927
Ym1	CAGGTCTGGCAATTCTTCTGAA	GTCTTGCTCATGTGTGTAAGTGA	NC_000069.6

### Statistical analysis

Statistics and graphing of numerical data were performed using GraphPad Prism7 software (GraphPad Software, La Jolla, CA, USA). Data are presented as means ± standard deviation (SD) from at least 3 independent biological replicates. Differences in the survival curves were assessed with Log-rank (Mantel-Cox) test. One-way ANOVA with Bonferroni post-tests was used to compare differences among more than two different groups and two-way ANOVA with Bonferroni post-tests was used to compare differences of parasitemia and change in body weight among more than two different groups. Pearson’s correlation coefficients were calculated to analyze correlations between the levels of galectins and cytokines. *P *< 0.05 was considered to be statistically significant.

## Results

### Prior *T. spiralis* infection decreased parasitemia of *Pb*ANKA-infected mice without affecting overall survival

**Fig. 1:**
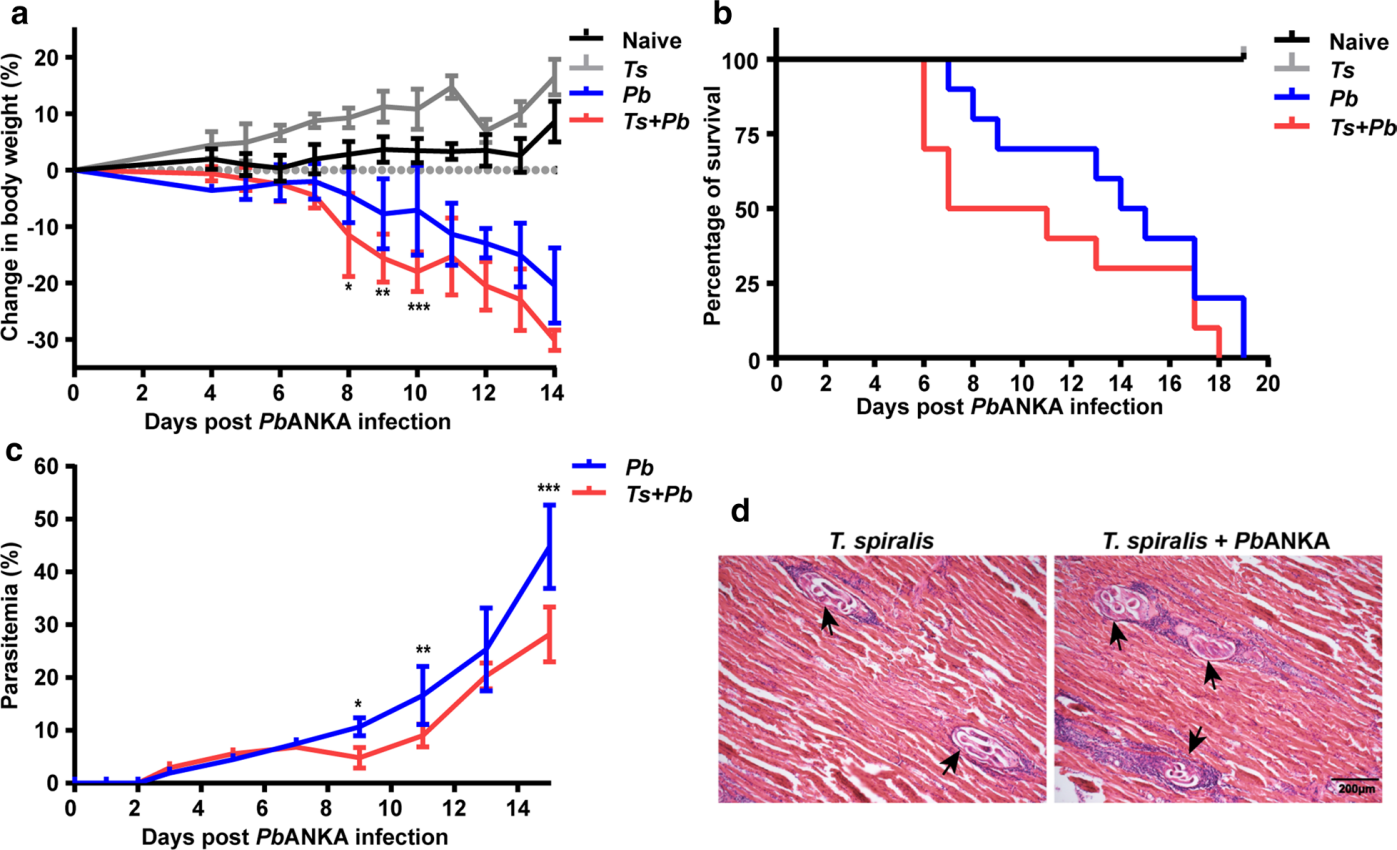
The change in body weight (**a**), survival rate (**b**), and time course of parasitemia (**c**) in different groups of mice. Kunming mice were infected with *Pb*ANKA alone or infected with *T. spiralis* and 9 days later were challenged with *Pb*ANKA. All the naive mice and *T. spiralis*-mono-infected mice survived during the observation period. *Pb*ANKA-mono-infected mice (*n* = 10) died between days 7 and 19 pi, *T. spiralis* + *Pb*ANKA-co-infected mice (*n* = 10) died between days 6 and 18 post-co-infection. The change in body weight and parasitemia are shown as the mean ± SD (*n* = 6 per group). *P < 0.05, **P < 0.01, and ***P < 0.001 *vs Pb*ANKA-mono-infected mice. Data are representative for two experiments. For evidence of *T. spiralis* infection (**d**), the histopathology of diaphragm stained with H&E is shown in a *T. spiralis*-mono-infected mouse and a *T. spiralis* + *Pb*ANKA-co-infected mouse on day 22 after *T. spiralis* infection. *Trichinella spiralis* encysted larvae were indicated by black arrows. The original magnification was 100× (*Scale-bar*: 200 µm)

To investigate whether prior *T. spiralis* infection alters the course of a subsequent malaria infection, we compared the changes in body weight, survival rate, and parasitemia of age-matched Kunming mice mono-infected with *Pb*ANKA or infected with *T. spiralis* and 9 days later challenged with *Pb*ANKA. Body weight were measured from day 4 following *Pb*ANKA infection, the results showed that *Pb*ANKA group and *T. spiralis + Pb*ANKA group had a similar tendency of body weight loss from days 7 to 14 pi. However, compared with *Pb*ANKA-mono-infection, significant weight loss was observed on days 8, 9, and 10 post-co-infection (two-way ANOVA: *F*_(1, 10)_ = 3.855, *P* = 0.022, *P* = 0.008, and *P *< 0.001, respectively) (Fig. 1a). Naive mice and *T. spiralis*-mono-infected mice all survived during the experiment. Mice of the *Pb*ANKA group died between 7 and 19 days pi, and approximately 50% of *Pb*ANKA-mono-infected mice succumbed between 7 and 14 days pi; while mice of the *T. spiralis + Pb*ANKA group died between 6 and 18 days, and approximately 50% of *T. spiralis + Pb*ANKA-co-infected mice succumbed between 6 and 11 days post-co-infection. However, there were no significant differences in overall survival between the two groups (Log-rank test: *χ*^2^ = 1.911, *df* = 1, *P* = 0.167) (Fig. 1b). Parasitemia was analyzed from day 3 after *Pb*ANKA infection. Although the levels of parasitemia were comparable for the *Pb*ANKA group and *T. spiralis* + *Pb*ANKA group from day 3 to 7 following *Pb*ANKA infection, the *Pb*ANKA group had significant higher percentages of infected erythrocytes than the *T. spiralis* + *Pb*ANKA group on days 9, 11, and 15 post-*Pb*ANKA infection (two-way ANOVA: *F*_(1, 10)_ = 2.283, *P* = 0.044, *P* = 0.003, and *P *< 0.001, respectively) (Fig. 1c). For evidence of *T. spiralis* infection, on day 22 after infection with 20 muscle larvae of *T. spiralis*, the diaphragm tissues of both *T. spiralis*-mono-infected and *T. spiralis* + *Pb*ANKA-co-infected mice were examined. Mice of both groups presented encysting larvae of *T. spiralis* in the skeletal muscles, which were surrounded by various inflammatory cells (Fig. 1d). Overall, the results demonstrated that prior *T. spiralis* infection decreases parasitemia levels but does not affect the survival outcome in a subsequent *Pb*ANKA infection.

### Prior *T. spiralis* infection increased liver and spleen indexes and liver pathology of *Pb*ANKA-infected mice

**Fig. 2:**
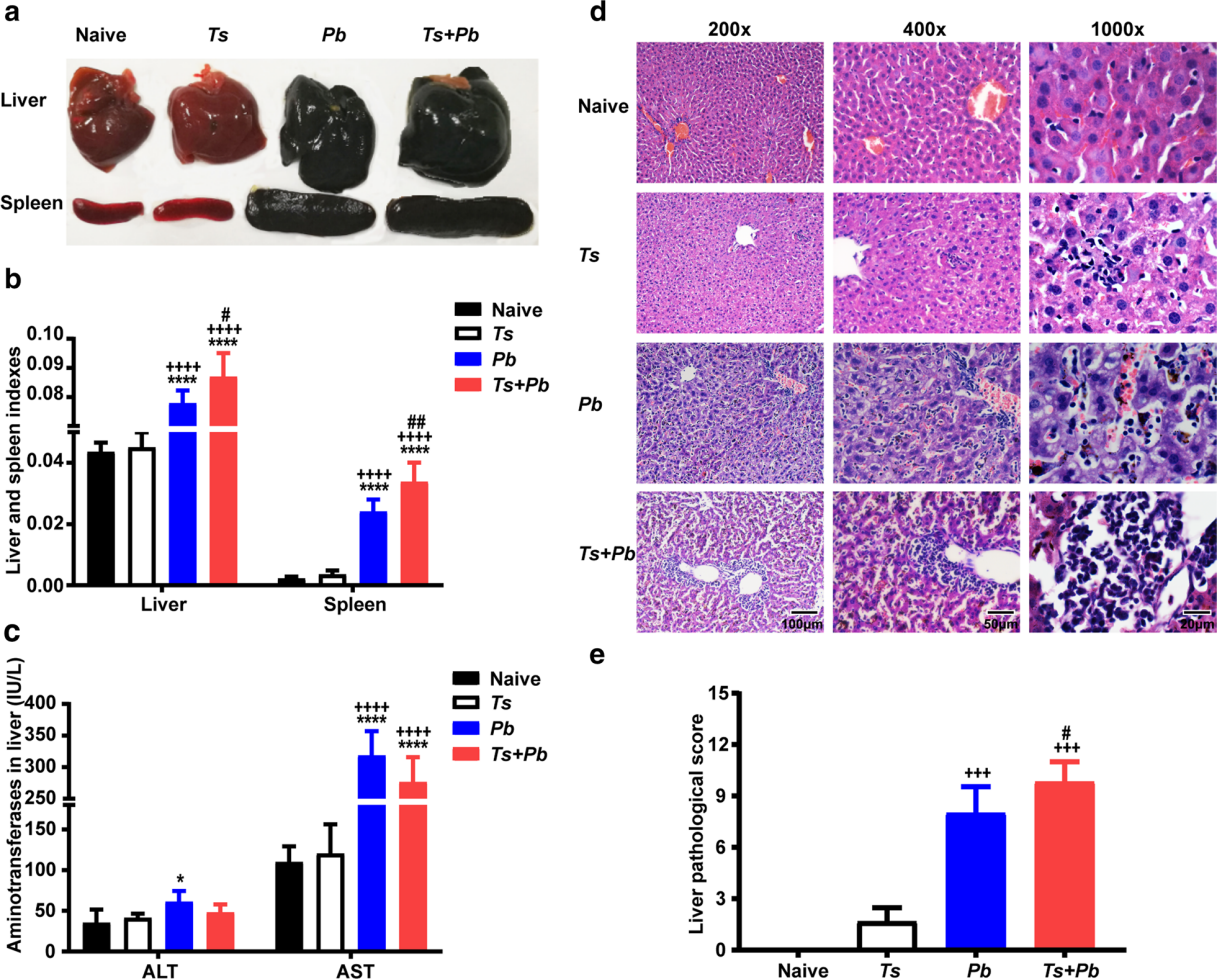
The gross specimens of liver and spleen (**a**), liver and spleen indexes (**b**), serum levels of AST and ALT (**c**), liver histopathology stained with H&E (**d**), and liver pathological semi-quantitation (**e**) of different groups. The measurements were performed on day 22 after *T. spiralis* infection and/or on day 13 after *Pb*ANKA infection. Shown are the liver of an uninfected mouse, a *T. spiralis*-mono-infected mouse, a *Pb*ANKA-mono-infected mouse, and a *T. spiralis* + *Pb*ANKA-co-infected mouse. Original magnifications were 200× (*Scale-bar*: 100 µm), 400× (*Scale-bar*: 50 µm), and 1000× (*Scale-bar*: 20 µm). Data are expressed as the mean ± SD (*n* = 6 per group). *P < 0.05 and ****P < 0.0001 *vs* naive mice; ^+++^*P* < 0.001 and ^++++^*P* < 0.0001 *vs T. spiralis*-mono-infected mice; ^*#*^*P *< 0.05 and ^*##*^*P *< 0.01 *vs Pb*ANKA-mono-infected mice

On day 22 after *T. spiralis* infection and/or on day 13 after *Pb*ANKA infection, the size of livers and spleens were notably different among the groups, hepatosplenomegaly was more marked in *T. spiralis + Pb*ANKA-co-infected mice compared with either infection separately (Fig. 2a). Liver and spleen indexes of different groups were determined by fixed calculation formula. Compared with naive mice or mice mono-infected with *T. spiralis*, there were significantly increased liver and spleen indexes in both *Pb*ANKA-mono-infected mice and *T. spiralis + Pb*ANKA-co-infected mice (one-way ANOVA: *F*_(3, 20)_ = 105.2 and *F*_(3, 20)_ = 103.8, respectively, *P* < 0.0001); whereas significantly increased liver and spleen indexes were found in co-infected mice compared with those of *Pb*ANKA-mono-infected mice (one-way ANOVA: *F*_(3, 20)_ = 105.2, *P* = 0.04 and *F*_(3, 20)_ = 103.8, *P* = 0.002, respectively) (Fig. 2b), suggesting that pre-*T. spiralis* infection regulates the immune response of the immune organs (such as liver and spleen) to *Plasmodium* parasite infection. We further investigated whether prior *T. spiralis* infection affects liver function of *Pb*ANKA-infected mice. Compared with naive mice or *T. spiralis*-mono-infected mice, there were significantly increased serum ALT in *Pb*ANKA-mono-infected mice (one-way ANOVA: *F*_(3, 20)_ = 4.333, *P* = 0.022), and significantly increased serum AST in both *Pb*ANKA-mono-infected and *T. spiralis + Pb*ANKA-co-infected mice (one-way ANOVA: *F*_(3, 20)_ = 37.8, *P* < 0.0001) (Fig. 2c). However, there were no significant differences in serum ALT and AST levels between the *Pb*ANKA group and the *T. spiralis + Pb*ANKA group, suggesting prior *T. spiralis* infection had no protective effect on liver function damage induced by malarial infection.

The livers of different groups of mice were examined histologically. On day 22 after *T. spiralis* infection and/or on day 13 after *Pb*ANKA infection, the liver tissues from naive mice showed no structural abnormalities. Acute small foci of inflammation were observed in the liver of mice with *T. spiralis*-mono-infection. However, the livers of both *Pb*ANKA and *T. spiralis + Pb*ANKA groups showed typical features of *Plasmodium* infection characterized by tissue edema, small necrotic foci, and malaria pigment deposition. Compared with *Pb*ANKA-mono-infected mice, there was more severe liver damage in co-infected mice, characterized by more malaria pigment deposition and inflammatory infiltration on day 13 post-co-infection (Fig. 2d). To evaluate liver damage after *Pb*ANKA infection, semi-quantitative liver inflammation scores based on liver pathological changes showed that compared with *T. spiralis*-mono-infected mice, there were significantly increased pathological scores in both the *Pb*ANKA group and the *T. spiralis + Pb*ANKA group (one-way ANOVA: *F*_(2, 15)_ = 74.55, *P *< 0.001); however, compared with *Pb*ANKA-mono-infected mice, there was a significantly increased pathological score in the liver of co-infected mice (one-way ANOVA: *F*_(2, 15)_ = 74.55, *P* = 0.049) (Fig. 2e), suggesting that prior *T. spiralis* infection resulted in accelerated liver pathology with a subsequent *Pb*ANKA infection.

### Prior *T. spiralis* infection increased the expression of Gal-1 and Gal-3 in the liver and spleen of *Pb*ANKA-infected mice

**Fig. 3:**
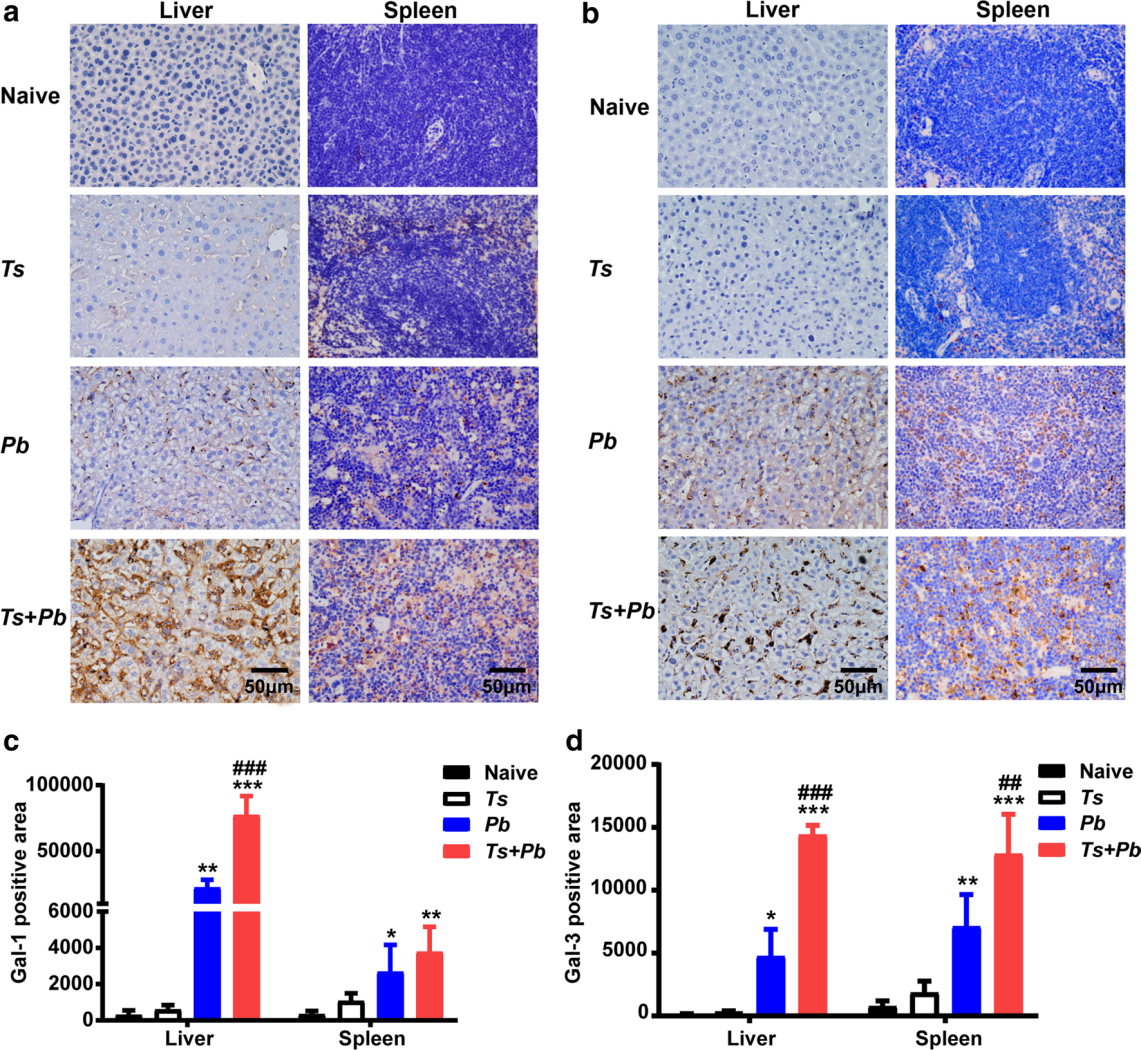
Co-infection with *T. spiralis* increased the expression of Gal-1 and Gal-3 in the liver and spleen of mice infected with *Pb*ANKA. Immunohistochemical expression of Gal-1 (**a**) and Gal-3 (**b**) and semi-quantitative analysis of the expression levels of Gal-1 (**c**) and Gal-3 (**d**) in the liver and spleen of different groups of mice on day 22 after *T. spiralis* infection and/or on day 13 after *Pb*ANKA infection. Shown are immunohistochemical staining for Gal-1 or Gal-3 in the liver and spleen of an uninfected mouse, a *T. spiralis*-mono-infected mouse, a *Pb*ANKA-mono-infected mouse, and a *T. spiralis* + *Pb*ANKA-co-infected mouse. The original magnification was 400× (*Scale-bar*: 50 µm). Data are presented as the mean ± SD (*n* = 6 per group). *P < 0.05, **P < 0.01, and ***P < 0.001 *vs* naive mice; ^##^*P *< 0.01 and ^###^*P *< 0.001 *vs Pb*ANKA-mono-infected mice

Compared with naive mice or *T. spiralis*-mono-infected mice, increased immunohistochemical expression of Gal-1 (Fig. 3a) and Gal-3 (Fig. 3b) were observed in the liver and spleen sections of both *Pb*ANKA-mono-infected and *T. spiralis* + *Pb*ANKA-co-infected mice. Semi-quantitative immunohistochemical analysis showed that compared with *Pb*ANKA-mono-infected mice, there were significantly increased expression levels of Gal-1 and Gal-3 in the liver (one-way ANOVA: *F*_(3, 20)_ = 86.93 and *F*_(3, 20)_ = 33.65, respectively, *P* < 0.001) and increased Gal-3 expression level in the spleen (one-way ANOVA: *F*_(3, 20)_ = 8.867, *P* = 0.002) of co-infected mice (Fig. 3c, d). The data provide evidence that Gal-1 and Gal-3 may be involved in the pathogenic basis of malaria.

### Prior *T. spiralis* infection increased CD68^+^ macrophage and neutrophil elastase expression levels in the liver and spleen of *Pb*ANKA-infected mice

**Fig. 4:**
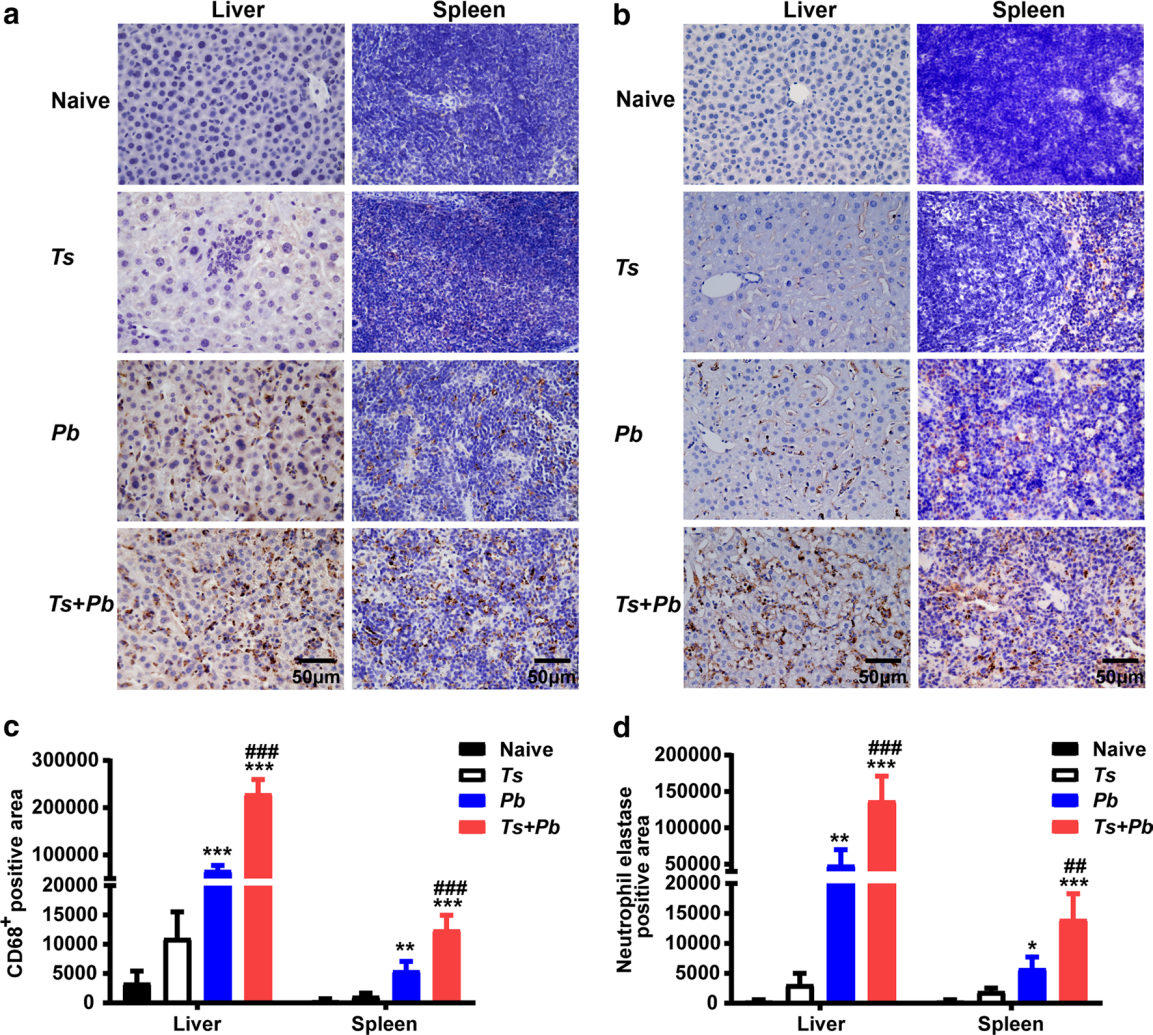
Co-infection with *T. spiralis* stimulated the infiltration of CD68^+^ macrophages and elastase-expressed neutrophils in the liver and spleen of mice infected with *Pb*ANKA. Immunohistochemical staining for CD68^+^ macrophages (**a**) and neutrophil elastase-positive cells (**b**), and semi-quantitative analysis of the expression levels of CD68^+^ macrophages (**c**) and neutrophil elastase (**d**) in the liver and spleen of different groups of mice on day 22 after *T. spiralis* infection and/or on day 13 after *Pb*ANKA infection. Shown are immunohistochemical staining for CD68^+^ macrophages or elastase-expressed neutrophils in the liver and spleen of an uninfected mouse, a *T. spiralis*-mono-infected mouse, a *Pb*ANKA-mono-infected mouse, and a *T. spiralis* + *Pb*ANKA-co-infected mouse. The original magnification was 400× (*Scale-bar*: 50 µm). Data are presented as means ± SD (*n* = 6 per group). *P < 0.05, **P < 0.01, and ***P < 0.001 *vs* naive mice; ^*##*^*P *< 0.01 and ^*###*^*P *< 0.001 *vs Pb*ANKA-mono-infected mice

Neutrophil elastase is a cytotoxic serine protease, which is stored in the azurophil granules of neutrophil granulocytes and is released by activated neutrophils. CD68^**+**^ macrophages and elastase-positive neutrophils were detected in the liver and spleen sections of both the *Pb*ANKA group and the *T. spiralis* + *Pb*ANKA group on day 13 post-*Pb*ANKA infection. Immunohistochemical staining showed that there were few CD68^**+**^ macrophages and no elastase-positive neutrophils observed in the liver and spleen of uninfected controls or the liver of *T. spiralis*-mono-infected mice. However, a large number of CD68^**+**^ macrophages (Fig. 4a) and elastase-positive neutrophils (Fig. 4b) were observed in the spleen of *T. spiralis*-mono-infected mice, and the liver and spleen of both *Pb*ANKA-mono-infected and *T. spiralis* + *Pb*ANKA-co-infected mice. Semi-quantitative analysis showed that compared with naive mice, there were significantly increased expression levels of CD68^**+**^ macrophage and neutrophil elastase in the liver (one-way ANOVA: *F*_(3, 20)_ = 167.8, *P *< 0.001 and *F*_(3, 20)_ = 41.41 *P* = 0.003, respectively) and spleen (one-way ANOVA: *F*_(3, 20)_ = 55.2, *P* = 0.002 and *F*_(3, 20)_ = 26.33, *P* = 0.032, respectively) of *Pb*ANKA-mono-infected mice, and significantly increased expression levels of CD68^+^ macrophage and neutrophil elastase in the liver (one-way ANOVA: *F*_(3, 20)_ = 167.8 and *F*_(3, 20)_ = 41.41, respectively, *P *< 0.001) and spleen (one-way ANOVA: *F*_(3, 20)_ = 55.2 and *F*_(3, 20)_ = 26.33, respectively, *P *< 0.001) of *T. spiralis* + *Pb*ANKA-co-infected mice. Compared with *Pb*ANKA-mono-infected mice, there were significantly increased expression levels of CD68^**+**^ macrophage and neutrophil elastase in the liver (one-way ANOVA: *F*_(3, 20)_ = 167.8 and *F*_(3, 20)_ = 41.41, respectively, *P *< 0.001) and spleen (one-way ANOVA: *F*_(3, 20)_ = 55.2, *P *< 0.001 and *F*_(3, 20)_ = 26.33, *P* = 0.002, respectively) of co-infected mice (Fig. 4c, d).

### Prior *T. spiralis* infection showed more eosinophils in the liver and increased levels of ECP and EPX in the spleen of *Pb*ANKA-infected mice

**Fig. 5:**
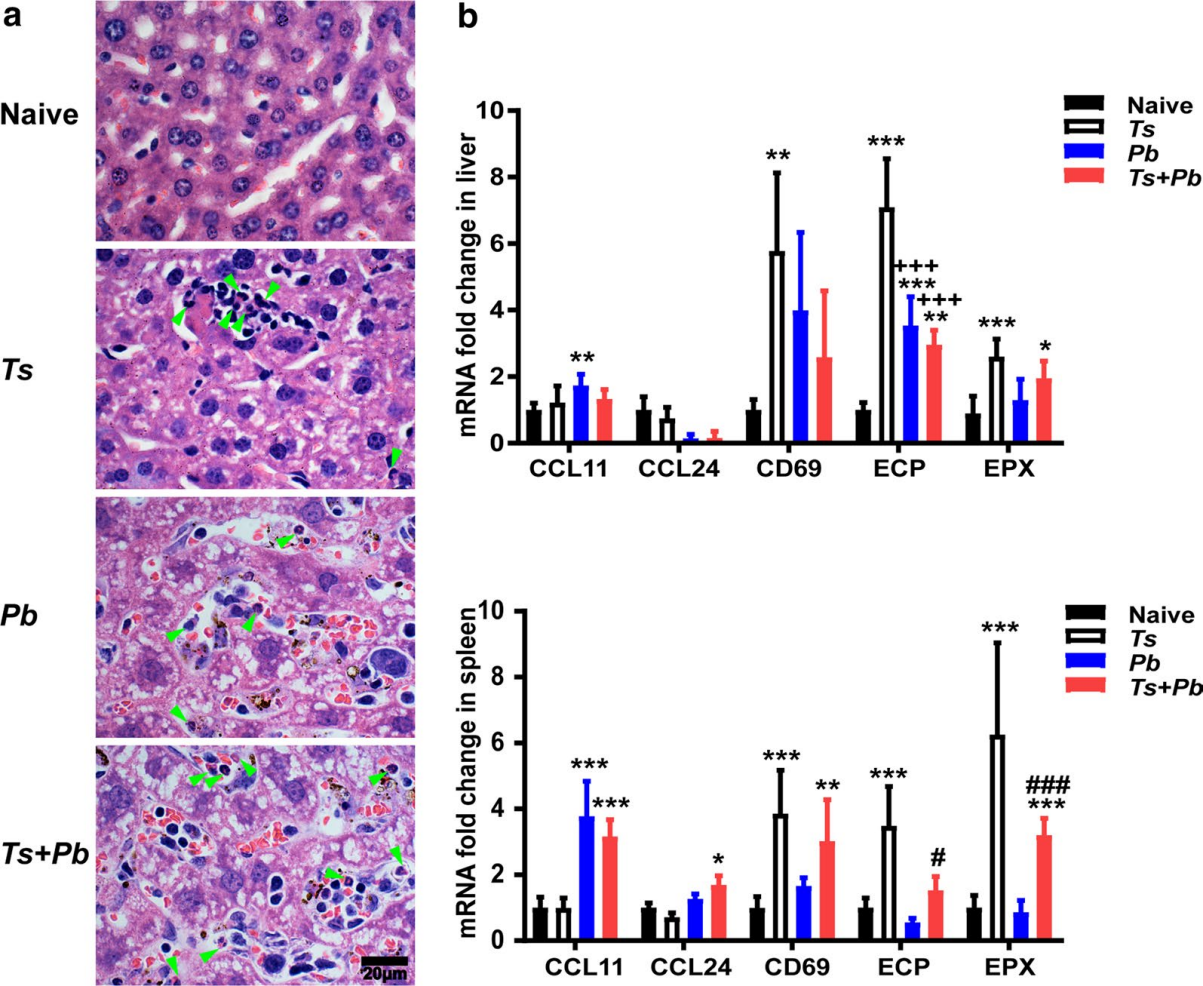
Eosinophils in the liver (**a**) and mRNA expression of CCL11, CCL24, CD69, ECP, and EPX detected by using qRT-PCR in the liver and spleen (**b**) of different groups of mice on day 22 after *T. spiralis* infection and/or on day 13 after *Pb*ANKA infection. Shown are H&E staining for eosnophils in the liver of an uninfected mouse, a *T. spiralis*-mono-infected mouse, a *Pb*ANKA-mono-infected mouse, and a *T. spiralis* + *Pb*ANKA-co-infected mouse. Eosinophils were indicated by green arrows. The original magnification 1000× (*Scale-bar*: 20 µm). Data are presented as the mean ± SD; there were 6 mice in each group and the data represents from two experiments. *P < 0.05, **P < 0.01, and ***P < 0.001 *vs* naive mice; ^+++^ *P *< 0.001 *vs T. spiralis*-mono-infected mice; ^#^*P *< 0.05 and ^###^ *P *< 0.001 *vs Pb*ANKA-mono-infected mice

By H&E staining, no eosinophils were observed in the liver of naive mice. However, on day 22 after *T. spiralis* infection and/or on day 13 after *Pb*ANKA infection, more eosinophils were observed in the livers of the *Pb*ANKA group, and even more eosinophils were observed in the livers of the *T. spiralis* group and the *T. spiralis + Pb*ANKA group (Fig. 5a). To determine whether the difference in liver damage was reflected in the activation status of eosinophils, liver and spleen from the *Pb*NAKA group or the *T. spiralis* + *Pb*ANKA group were analyzed for the expression of eotaxin (CCL-11 and CCL-24), ECP, and EPX. Compared with naive mice, there was significantly increased CCL11 levels in the liver (one-way ANOVA: *F*_(3, 20)_ = 9.059, *P* = 0.009) and spleen (one-way ANOVA: *F*_(3, 20)_ = 32.24, *P *< 0.001) of *Pb*ANKA-mono-infected mice, and significantly increased levels of CCL11 and CCL24 in the spleen (one-way ANOVA: *F*_(3, 20)_ = 32.24, *P* < 0.001 and *F*_(3, 20)_ = 3.044, *P* = 0.045, respectively) of *T. spiralis* + *Pb*ANKA-co-infected mice. Compared with naive mice, there were significantly increased ECP levels in the livers (one-way ANOVA: *F*_(3, 20)_ = 49.86, *P *< 0.001, *P *< 0.001, and *P* = 0.005, respectively) of *T. spiralis*-mono-infected, *Pb*ANKA-mono-infected, and co-infected mice, and in the spleen (one-way ANOVA: *F*_(3, 20)_ = 23.51, *P *< 0.001) of *T. spiralis*-mono-infected mice, significantly increased EPX level in the liver and spleen of *T. spiralis*-mono-infected mice (one-way ANOVA: *F*_(3, 20)_ = 11.34 and *F*_(3, 20)_ = 18.43, respectively, *P *< 0.001), and significantly increased EPX level in the liver (one-way ANOVA: *F*_(3, 20)_ =  = 11.34, *P* = 0.012) and spleen (one-way ANOVA: *F*_(3, 20)_ = 18.43, *P *< 0.001) of co-infected mice. Compared with *Pb*ANKA-mono-infected mice, there were significantly increased levels of ECP (one-way ANOVA: *F*_(3, 20)_ = 23.51, *P* = 0.023) and EPX (one-way ANOVA: *F*_(3, 20)_ = 18.43, *P *< 0.001) in the spleen of co-infected mice (Fig. 5b). The data indicate that prior *T. spiralis* infection does significantly increase eosinophil activation, which may have an effect on liver pathology of mice with a subsequent *Pb*ANKA infection.

### Prior *T. spiralis* infection promoted the expression of Gal-1, Gal-3, IFNγ, IL-6, IL-1β, iNOS, and Ym1 in the liver, spleen, or peritoneal macrophage of *Pb*ANKA-infected mice

**Fig. 6:**
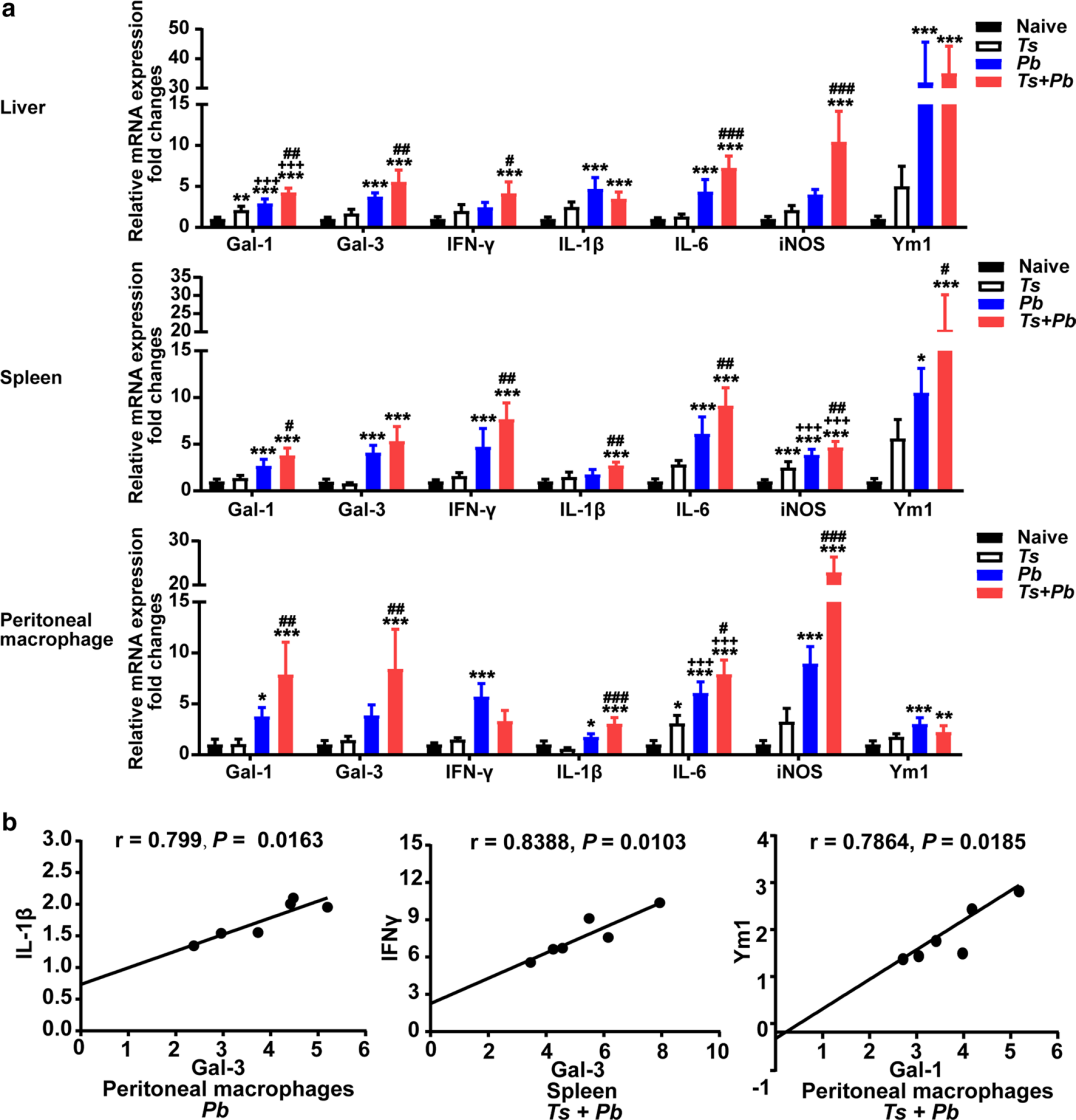
**a** The mRNA expression of Gal-1, Gal-3, IFNγ, IL-1β, IL-6, iNOS, and Ym1 detected by using qRT-PCR in the liver, spleen, and peritoneal macrophages of different groups of mice on day 22 after *T. spiralis* infection and/or on day 13 after *Pb*ANKA infection. Data are presented as the mean ± SD (*n* = 6 per group). *P < 0.05, **P < 0.01, and ***P < 0.001 *vs* naive mice; ^+++^*P *< 0.001 *vs T. spiralis*-mono-infected mice; ^*#*^*P *< 0.05, ^*##*^*P *< 0.01, and ^*###*^*P *< 0.001 *vs Pb*ANKA-mono-infected mice. **b** Significant correlations were found between Gal-3 and IL-1β in the peritoneal macrophages from *Pb*ANKA-mono-infected mice, between Gal-3 and IFNγ in the spleen of *T. spiralis* + *Pb*ANKA-co-infected mice, and between Gal-1 and Ym1 in the peritoneal macrophages from co-infected mice. Data are expressed as the mean ± SD (*n* = 6 per group). The *r* value generates the theoretical line of best fit, and the *P*-value indicates the significance of the correlation

To determine the immune responses of the livers, spleens, and macrophages of different groups of mice, the mRNA expression of Gal-1, Gal-3, IFNγ, IL-1β (M1 marker), IL-6, iNOS (M1 marker), and Ym1 (M2 marker) in the peritoneal macrophage from different groups were measured on day 22 after *T. spiralis* infection and/or on day 13 after *Pb*ANKA infection. Compared with *Pb*ANKA-mono-infected mice, there were significantly increased expression levels of Gal-1 (one-way ANOVA: *F*_(3, 20)_ = 48, *P* = 0.004), Gal-3 (one-way ANOVA: *F*_(3, 20)_ = 36.64, *P* = 0.006), IFNγ (one-way ANOVA: *F*_(3, 20)_ = 13.06, *P* = 0.016), IL-6 (one-way ANOVA: *F*_(3, 20)_ = 45.25, *P *< 0.001), and iNOS (one-way ANOVA: *F*_(3, 20) _= 28.49, *P *< 0.001) in the liver; significantly increased expression levels of Gal-1 (one-way ANOVA: *F*_(3, 20)_ = 29.87, *P* = 0.015), IFNγ (one-way ANOVA: *F*_(3, 20)_ = 31.65, *P* = 0.005), IL-1β (one-way ANOVA: *F*_(3, 20)_ =  = 16.43, *P* = 0.004), IL-6 (one-way ANOVA: *F*_(3, 20)_ = 42.15, *P* = 0.005), iNOS (one-way ANOVA: *F*_(3, 20)_ = 105.4, *P* = 0.009), and Ym1 (one-way ANOVA: *F*_(3, 20)_ = 16.19, *P* = 0.015) in the spleens, and significantly increased expression levels of Gal-1 (one-way ANOVA: *F*_(3, 20)_ = 21.96, *P* = 0.002), Gal-3 (one-way ANOVA: *F*_(3, 20)_ = 16.66, *P* = 0.004), IL-1β (one-way ANOVA: *F*_(3, 20)_ = 46.2, *P *< 0.001), IL-6 (one-way ANOVA: *F*_(3, 20)_ = 56.11, *P* = 0.024), and iNOS (one-way ANOVA: *F*_(3, 20)_ = 134.6, *P *< 0.001) in the peritoneal macrophages of *T. spiralis + Pb*ANKA-co-infected mice (Fig. 6a). The data indicate that prior *T. spiralis* infection markedly increases Gal-1, Gal-3, and cytokine responses in the livers, spleens, and peritoneal macrophages of mice induced by malaria infection.

### Correlations between Gal-1/Gal-3 and IFNγ, IL-1β, IL-6, iNOS, or Ym1 in the liver, spleen, and peritoneal macrophages of different groups

The correlations between mRNA levels of Gal-1/Gal-3 and IFNγ, IL-1β, or Ym1 in the liver, spleen, and peritoneal macrophages of different groups of mice were analyzed, only significant correlations are provided here. Significant correlations were found between Gal-3 and IL-1β (Pearson’s correlation: *r* = 0.799, *P* = 0.0163) in the peritoneal macrophages from *Pb*ANKA-mono-infected mice on day 13 after infection, between Gal-3 and IFNγ (Pearson’s correlation: *r* = 0.8388, *P* = 0.0103) in the spleen of *T. spiralis* + *Pb*ANKA-co-infected mice on day 13 after co-infection, and between Gal-1 and Ym1 (Pearson’s correlation: *r* = 0.7864, *P* = 0.0185) in the peritoneal macrophages from co-infected mice on day 13 after co-infection (Fig. 6b).

## Discussion

Malaria caused by *Plasmodium* spp. is a major health problem in many countries in the tropical and subtropical regions of the world. Hepatocellular dysfunction is recognized as one of the clinical features in severe malaria [23]. However, the mechanism of hepatic injury during malaria is still unknown. Helminths exert strong immunomodulatory effects in their hosts [24]. In endemic regions, it is not uncommon for patients to be co-infected with helminths and malaria. A number of studies from different continents have shown complex interactions between different gastrointestinal nematodes (such as *A. lumbricoides*, *T. trichiura*, and hookworm) and malaria, with different immunomodulatory properties or conflicting consequences [6–8, 25]. *Trichinella spiralis* infection has been identified to have strong immunomodulatory effects [26]. Mice challenged with *Listeria monocytogenes* at 7 or 21 days after *T. spiralis* infection showed a higher mean lethal dose and longer survival time than control mice [27]. Infection with *T. spiralis* activates macrophages and mediators released by activated macrophages participate in the non-specific immunity [18]. So far, the effect of prior *T. spiralis* infection on *P. berghei*-induced liver disease has not been reported. To test the hypothesis that a pre-existing Th2 environment alters the general course of a subsequent malaria infection, in the present study, we compared the physiological and immunopathological responses of age-matched Kunming mice mono-infected with *Pb*ANKA or infected with *T. spiralis* and nine days later challenged with *Pb*ANKA. Our data demonstrated that *T. spiralis* + *Pb*ANKA-co-infection results in statistically significant weight loss in only three days but markedly decreased parasitemia; however, the overall survival durations had no difference compared with *Pb*ANKA-mono-infected mice. It has been reported that children even with a light *S. haematobium* infection presented lower *P. falciparum* parasite densities than children not infected with *S. haematobium* [28]. However, mice co-infected with *S. mansoni* and *P. chabaudi* AS develop increased malaria parasitemia [29].

It has been reported that hepatosplenomegaly is more frequent in severe malaria [30]. Young children during malaria attacks have lymphocyte infiltrations in hepatic sinusoids, hyperplasia of Kupffer cells, and deposits of malaria pigment [31]. The reticular endothelial system of the liver is able to eliminate parasite-derived hemozoin and *Plasmodium*-infected erythrocytes through phagocytosis [32]. Exacerbated hepatosplenomegaly has been shown among Kenyan school children, living in a malaria and schistosomiasis co-transmission area [33, 34]. Hepatosplenomegaly caused by chronic exposure to malaria is clearly associated with increased circulating levels of pro-inflammatory mediators amongst *S. mansoni-*infected children [34]. Animal experiments also demonstrated that hepatosplenomegaly in BALB/c mice was much higher in the context of schistosome-malaria co-infection compared with infection of either *P. yoelii* or *S. mansoni* alone [35]. ALT and AST are primarily expressed in liver cells, when the physiological and biochemical function and the integrity of hepatocytes are damaged, resulting in elevated serum AST and ALT levels [36]. In the present study, compared with *Pb*ANKA-mono-infected mice, more severe hepatosplenomegaly and more overt hepatic pathology with increased hepatic inflammatory infiltrates of CD68^**+**^ macrophages, elastase-positive neutrophils, and eosinophils were measured, but similar serum levels of liver function enzymes ALT and AST were examined in the co-infected mice on day 13 post-*Pb*ANKA infection.

Peripheral blood mononuclear macrophages can be generally divided into classically activated macrophages (M1) and alternatively activated macrophages (M2). M1 macrophages, activated by Th1 cytokines of the infected host, upregulate IL-12 and iNOS expressions. In contrast, M2 macrophages, driven by the Th2 cytokines IL-4 and IL-13, express high levels of arginase-1, Ym1, mannose receptor, and resistance-like molecule-α [37, 38]. Tissue resident macrophages play key roles in infection with either *Pb*ANKA [20] or *T. spiralis* [39]. Prior *Nippostrongylus brasiliensis* infection has a lasting effect on lung macrophages, where *Pb*ANKA-induced M1-like response was reduced by previous M2 polarization [40]. Kupffer cell phagocytic activity in liver is markedly increased in rats with a high parasitemic load of malarial *P. berghei* infection [41], and electron microscopy revealed that Kupffer cells fill with damaged RBCs, pRBCs, and haemoglobin degradation pigment [42]. In the present study, mice were infected with *T. spiralis* and nine days later were challenged with *Pb*ANKA, and the functional characteristics of macrophages induced by prior *T. spiralis* infection in the regulation of immune reaction and inflammation in co-infected mice were examined. The ability of *T. spiralis* infection to modulate macrophage activation was determined by detecting the production of effector molecules including gelactins, iNOS, Ym1, and cytokines. Interestingly, CD68^+^ macrophage expression levels were significantly increased in the liver and spleen of co-infected mice compared with *Pb*ANKA-mono-infected mice. There were significantly increased expression levels of Gal-1, Gal-3, IL-1β, IL-6, and iNOS in the peritoneal macrophage isolated from co-infected mice than that isolated from *Pb*ANKA-mono-infected mice. It is worth noting that M1 macrophage markers were increased in the liver, spleen, or peritoneal macrophages of co-infected mice, suggesting that the immune system of co-infected mice developed a M1 polarization of macrophages with an emerging predominance of IL-1β, IL-6, and iNOS response, which may contribute to the liver damage of co-infected mice. However, it has been reported that increased Gal-3 expression and secretion is a feature of M2 macrophage activation [43].

Neutrophil activation and circulating neutrophil extracellular traps are elevated in human malaria, which may contribute to pathogenesis of severe falciparum malaria [44]. Neutrophil activation is also associated with pathogenesis of experimental cerebral malaria and acute lung injury/acute respiratory distress syndrome in murine models [45, 46]. An *in vitro* study demonstrated that neutrophils can kill *P. falciparum* parasites [47]. In addition, after transferring neutrophils obtained from the spleens of *Pb*ANKA-infected adult rats to young rats, they exerted a significant dose-dependent anti-parasite effect, demonstrating the role of neutrophils and their effector proteins in the control of early phase blood *Plasmodium* parasite growth [48]. The immune response of long-lasting infection of muscles with *Trichinella* is mainly characterized by a Th2 phenotype [49]. The present study showed that compared with *Pb*ANKA-mono-infected mice, there were significantly increased neutrophil elastase expression levels in the liver and spleen of *T. spiralis + Pb*ANKA-co-infected mice, indicating neutrophils may have detrimental effects on either controlling malaria parasites or aggravating liver pathology during the co-infection.

It has been reported that an eosinophilia is induced in *P. falciparum* malaria and the concentrations of eosinophil-derived granule proteins (ECP and EPX) are higher in patients with cerebral malaria than in uncomplicated cases or cases of severe malarial anemia, indicating eosinophil granule proteins are important in both the control of malaria infection and pathogenesis of severe malaria [50]. An *in vitro* study showed that the secreted products from eosinophils can kill *P. falciparum* parasites [51], but eosinophils may also contribute to pathology by release of granule proteins such as ECP and EPX/eosinophil-derived neurotoxin [52]. Eotaxin is a member of the CC chemokine family, including three subfamilies, e.g. CCL11/eotaxin-1 [53], CCL24/eotaxin-2 [54], and CCL26/eotaxin-3 [55]. Chemokines CCL11 and CCL24 produced in the liver following halothane treatment play a role in attracting eosinophils to the liver [56]. *Trichinella spiralis* induces a pronounced eosinophilia that coincides with establishment of larval stages in skeletal muscles [57], and eosinophils served to limit the number of newborn larvae of *T. spiralis* that migrated in the tissues and colonized the skeletal muscles [58]. Eosinophils, as crucial to immune regulation, support the survival of muscle larvae of *T. spiralis* [57]; meanwhile eosinophils can be toxic for host tissues [59]. In the present study, we observed that eosinophils were increased in the liver of *T. spiralis* + *Pb*ANKA-co-infected mice on day 13 after co-infection. Measured *via* qRT-PCR, the mRNA levels of ECP and EPX were significantly increased in the spleen of co-infected mice compared to *Pb*ANKA-mono-infected mice, indicating that eosinophil and eosinophil granule proteins may play an important role in the pathogenisis of *T. spiralis*-malaria concurrent infection.

Galectins are a family of lectins, which contain conserved carbohydrate-recognition domains for β-galactosides [60]. Host galectins have been shown to modulate the effector function of mast cells, neutrophils, and eosinophils [61]. Gal-1 modulates protective CD4^+^ T cell responses during acute hepatic *Leishmania donovani* infection by limiting IFNγ-producing CD4^+^ T cell numbers [62]. Gal-1 can induce human neutrophil migration [63], reduce eosinophil recruitment to the airways in a mouse model [64], and modulate macrophage polarization and protein secretion in an *in vitro* study [65]. Soluble Gal-1 enhances T cell IL-10 production and decreases cytotoxic T cell survival, reduces macrophage responses to IFNγ [66], and promotes an M2-like macrophage phenotype [67]. Gal-1 immunoregulatory function is shown to contribute to enhanced parasite control, survival, and Th1 effector function in Gal-1-deficient mice infected with *Trypanosoma cruzi* [68]. Gal-3 is abundantly expressed and secreted by macrophages [69]. It has been demonstrated that alternative macrophage activation induced by extracellular Gal-3 *via* IL-4/IL-13 is repressed in Gal-3-deficient mice [43]. Gal-3-deficient mice are partially protected against experimental cerebral malaria caused by *Pb*ANKA infection but developed higher peripheral parasitemia [70]. Studies also demonstrated that Gal-3 facilitates neutrophil activation [71, 72], and Gal-3 bound to the cell surface of eosinophils is essential for eosinophil trafficking under flow and migration [73]. Gal-3 regulates the capacity of dendritic cells to support NKT-cell-mediated liver injury, playing an important pro-inflammatory role in acute liver injury [74]. The immunoregulatory role of Gal-1 and Gal-3 is relevant for the induction of the host response during liver invasion by *Entamoeba histolytica* [75]. So far there have been no reports regarding the role of Gal-1 in malaria. Our findings demonstrated that co-infection resulted in higher levels of mRNA levels of Gal-1, Gal-3, IFNγ, IL-6, and iNOS in the liver of co-infected mice compared with *Pb*ANKA-mono-infection. Since co-infected mice displayed inflammatory infiltrates and severe pathology of the liver, which indicates that Gal-1 and Gal-3 may have effects on both parasitemia control and hepatic injury. In addition, compared with *Pb*ANKA-mono-infection, co-infection resulted in elevated Gal-1, Gal-3, M1 markers (IL-1β and iNOS), and IL-6 in the peritoneal macrophages, which may be associated with enhanced liver immunopathology during the co-infection. It has been reported that inflammatory cytokines IFNγ and IL-6, which have elevated expression locally in the brain and systemically in the plasma, may represent surrogate markers for neutrophil-mediated response [76]. Gal-1 and Gal-3 are important mediators of inflammation, and both have the capacity to induce a respiratory burst in neutrophils. The reactive oxygen species produced may be destructive to the invading microorganisms as well as to the surrounding host tissue, indicating the possible role of galectins not only in defense toward infection but also in inflammatory-induced tissue destruction [77]. Notably, in the present study, significant correlations were found between Gal-3 and IL-1β in the peritoneal macrophages from *Pb*ANKA-mono-infected mice and between Gal-1 and Ym1 in the peritoneal macrophages and between Gal-3 and IFNγ in the spleen of *T. spiralis* + *Pb*ANKA-co-infected mice. It has been reported that Gal-3 as an amplifier of IL-1β responses exacerbates the response to IL-1β by stimulating the secretion of inflammatory cytokines [78]. Our data suggest that Gal-1 and/or Gal-3 play a regulatory role during malaria infection or during the co-infection with *T. spiralis*.

## Conclusions

This study demonstrated that *Pb*ANKA infection is associated with an increased immune responsiveness that is affected by prior *T. spiralis* infection. Concurrent nematode infection strongly modulates multiple aspects of host immunity to blood-stage malaria, including immune cell function, cytokine production, and galectin responses. Consequently, the development of immunity to malaria is increased, leading to significantly decreased malaria parasitemia, exacerbated malaria-induced liver immunopathology, accompanied with elevated expression levels of Gal-1 and Gal-3, increased activation of macrophages, neutrophils, and eosinophils and increased production of their mediators in co-infected mice. Furthermore, our data demonstrated that a pre-existing helminth infection aggravates the secondary malaria infection, thus the control of helminth infection in a malaria epidemic area is of critical importance. Further study is needed to clarify the exact role of Gal-1 and Gal-3 in the pathogenesis of helminth-malaria concurrent infections.

## Data Availability

Data supporting the conclusions of this article are included within the article.

## Abbreviations

ALT: Alanine aminotransferase
AST: Aminotransferase
CCL: C-C motif chemokine ligand
ECP: Eosinophil cationic protein
EPX: Eosinophil protein X
Gal: Galectin
H&E: Hematoxylin and eosin
IFNγ: Interferon-gamma
IL: Interleukin
iNOS: Inducible nitric oxide synthase
ip: Intraperitoneal
*Pb*ANKA: *Plasmodium berghei* of strain ANKA
PBS: Phosphate buffered solution
Pi: Post-infection
pRBCs: Parasitized red blood cells
qRT-PCR: Quantitative real-time reverse transcription polymerase chain reaction
SD: Standard deviation
